# Time and Concentration Dependent Effects of Short Chain Fatty Acids on Lipopolysaccharide- or Tumor Necrosis Factor α-Induced Endothelial Activation

**DOI:** 10.3389/fphar.2018.00233

**Published:** 2018-03-19

**Authors:** Meng Li, Betty C. A. M. van Esch, Paul A. J. Henricks, Johan Garssen, Gert Folkerts

**Affiliations:** ^1^Division of Pharmacology, Utrecht Institute for Pharmaceutical Sciences, Faculty of Science, Utrecht University, Utrecht, Netherlands; ^2^Immunology, Nutricia Research, Utrecht, Netherlands

**Keywords:** SCFA, endothelial activation, anti-inflammatory effects, adhesion molecules, cytokines

## Abstract

**Background and Aim:** Endothelial activation is characterized by excessive production of cytokines and chemokines as well as adhesion molecules expression which is involved in the development of atherosclerosis. The aim of our study is to investigate the effects of short chain fatty acids (SCFA) on lipopolysaccharide (LPS) or tumor necrosis factor alpha (TNFα)-induced endothelial activation.

**Methods and Results:** Human umbilical vein endothelial cells (HUVEC) were pre-treated with acetate (10 mM), butyrate (0.1 mM) or propionate (0.3 mM) for 1, 16, or 24 h and then stimulated with LPS (1 or 10 μg/ml) or TNFα (100 pg/ml or 1 ng/ml) for 6, 12, or 24 h. Cytokines in the supernatant were measured by ELISA. HUVEC were pre-treated with acetate (10 mM), butyrate (5 mM) or propionate (10 mM) for 24 h and then stimulated with LPS (1 μg/ml) or TNFα (1 ng/ml) for 8 h. The expression of the adhesion molecules intracellular adhesion molecule-1 (ICAM-1) and vascular cell adhesion molecule-1 (VCAM-1) was detected by flow cytometry. The human blood mononuclear cell adhesive level to HUVEC monolayer was measured. LPS and TNFα induced a significant increase in the release of interleukin-6 (IL-6) and IL-8. Acetate, butyrate and propionate reduced IL-6 and IL-8 levels and the magnitude was dependent on the incubation times. LPS or TNFα increased ICAM-1 and VCAM-1 expression. Pre-incubation with acetate had no effect. In contrast, butyrate and propionate decreased VCAM-1 expression in TNFα stimulated cells but showed no effects on ICAM-1 expression. Butyrate significantly inhibited the adhesion of mononuclear cells to an endothelial monolayer and propionate was less effective.

**Conclusion:** SCFA, including acetate, butyrate and propionate, influenced LPS- or TNFα-induced endothelial activation by inhibiting the production of IL-6 and IL-8, and reducing the expression of VCAM-1 and subsequent cell adhesion. Results were dependent on the concentrations and pre-incubation time of each SCFA and stimulation time of LPS or TNFα.

## Introduction

Vascular endothelial cells serve as an important barrier to sustain homeostasis by maintaining normal vascular tone and blood fluidity and low production and release of pro-/anti-inflammatory factors. The homeostasis is disturbed by inflammation which leads to endothelial activation (Shoda et al., 2016). Endothelial activation is a well-established early step to cardiovascular risk factors and is involved in the development of atherosclerosis (Favero et al., 2014). Endothelial activation induced by lipopolysaccharide (LPS, endotoxin) and pro-inflammatory cytokines such as tumor necrosis factor alpha (TNFα) is characterized by increased inflammatory cytokine production and adhesion molecule expression (Hunt and Jurd, 1998) which are two important steps in the development of atherosclerosis (Yan et al., 2015). Increased production of cytokines, such as interleukin-6 (IL-6) and IL-8, is regarded as diagnostic markers in inflammatory diseases including atherosclerosis, obesity, heart diseases, and sepsis (Kessler et al., 1999; Arnson et al., 2013; Cheng et al., 2013; Odegaard et al., 2016). IL-6 has broad impacts on immune cells as a lymphocyte-stimulating factor and is involved in innate and adaptive immunity (Hunter and Jones, 2015). IL-6 has pro- and anti-inflammatory effects which is dependent on the receptor that IL-6 binds to and IL-6 exerts pro-inflammatory effects on endothelial cells via binding to sIL-6R (Scheller et al., 2011). Therefore, in our study, IL-6 induced by LPS or TNFα is regarded as a pro-inflammatory factor. IL-8 is a chemokine secreted by activated macrophages and endothelial cells which stimulates vascular adhesion of neutrophils (Arnson et al., 2013; Zhang et al., 2014). Leukocyte transmigration over the endothelium is a multi-step process including capturing, rolling, leukocyte arrest, crawling to sites of exit and transmigration (Vestweber, 2015) and each step is regulated by distinct endothelial adhesion receptors such as intercellular adhesion molecule-1 (ICAM-1), vascular cell adhesion molecule-1 (VCAM-1) and selectins. Excessive expression of ICAM-1 and VCAM-1 is regarded as an early event of atherosclerosis (Zhang et al., 2013). The transmigrated inflammatory cells produce more cytokines and form a perpetual cycle between cytokines and cell adhesion. Therefore, attenuation of endothelial activation is a crucial step in preventing and treating cardiovascular diseases including atherosclerosis.

Unbalanced diets may lead to acute or chronic low-grade inflammation which is a risk factor for various pathological conditions including cardiovascular diseases. Based on epidemiological studies, increased consumption of dietary fibers decreases the risk of cardiovascular diseases, such as atherosclerosis, and this may be due to the enhanced production of short chain fatty acids (SCFA) (Threapleton et al., 2013). SCFA, such as acetate, butyrate and propionate, are the major products formed during colonic fermentation of dietary fibers. SCFA are used by local enterocytes as an energy resource or are transported across the gut epithelium into the bloodstream where they can interact with peripheral cells and tissues as signaling molecules. Under physiological condition, the concentrations of SCFA in the peripheral blood are very low due to the hepatic metabolism and the predominant SCFA in circulation is acetate (Peters et al., 1992). However, in *in vivo* experiments, administration of the pro-drug of butyrate (Edelman et al., 2003) or SCFA intravenously (McAndrew et al., 1999) increases the concentrations of SCFA into millimolar levels in blood and could be clinically used. SCFA, especially butyrate, were originally studied as anti-cancer agents, such as in colorectal cancer (Encarnacao et al., 2015). In recent researches, SCFA showed promising potential therapeutic effects in inflammatory bowel diseases (Scaldaferri et al., 2013), obesity, insulin resistance (Priyadarshini et al., 2016) and cardiovascular diseases (Threapleton et al., 2013). However, there are no studies about the effects of SCFA on LPS or TNFα-induced IL-6 and IL-8 production by primary human umbilical vein endothelial cells (HUVEC) and their roles in adhesion molecule expression are contradictory (Zapolska-Downar et al., 2004; Miller et al., 2005; Inoue et al., 2006). Therefore, we investigated the effect of SCFA (acetate, butyrate, and propionate) on LPS or TNFα-induced endothelial activation by regulation the production of IL-6 and IL-8 and expression of ICAM-1 and VCAM-1 under different experimental conditions.

## Materials and Methods

### Reagents and Materials

Sodium butyrate, propionate, and LPS (*Escherichia coli* 0111:B4) were purchased from Sigma-Aldrich, St. Louis, MO, United States. Acetate was bought form Merck Millipore (Germany). Cell cytotoxicity detection kit (lactate dehydrogenase, LDH) was obtained from Roche (Switzerland). Human IL-6 and IL-8 ELISA (enzyme-linked immunosorbent assay) kits were purchased from Invitrogen (Netherlands). Human recombinant TNFα, anti-human CD54 (ICAM-1) PE, anti-human CD106 (VCAM-1) PE and viability fixable dyes were bought from eBioscience (Netherlands). EGM-2 Bulletkit was bought from Lonza (Switzerland).

### Cell Culture and Treatment

Human umbilical vein endothelial cells from umbilical vein were provided by Mrs. J.H. van Kats-Renaud (University Medical Center, Utrecht) after informed consent and isolated and cultured by adapting the method of Jaffe et al. (1973). HUVEC were cultured in EGM-2 (Lonza) containing 2% fetal bovine serum and VEGF for rapid proliferation, and maintained in a humidified incubator at 37°C in 5% CO_2_. Medium was changed every 2–3 days. HUVEC were cultured until about 85% confluence and cells of passages 2–7 were used for the experiments.

#### LPS or TNFα Stimulated-HUVEC

To induce an inflammatory response in the HUVEC, two different triggers were used namely LPS and TNFα. The first goal was to find the optimal time and dose of stimulation and to investigate which cytokines were produced. HUVEC were incubated in LPS (1 or 10 μg/ml) and TNFα (100 pg/ml or 1 ng/ml) for 6, 12, and 24 h.

#### SCFA Treated LPS or TNFα-Stimulated HUVEC

Each SCFA was dissolved in EGM-2 and then was filtered over a 0.2 μm filter to make it sterile. The stock concentration of each SCFA was 10 times of final concentration and was stored in 4°C. Confluent HUVEC were pre-incubated with pre-warmed stock solution of each SCFA with final concentration of acetate (0.3, 1, 3, 10, 30, or 100 mM), butyrate (0.1, 0.3, or 1 mM) and propionate (0.1, 0.3, 1, or 5 mM) or medium only for 1, 16, or 24 h. Next, LPS at 1 or 10 μg/ml, TNFα at 100 pg/ml or 1 ng/ml or medium only were added for 6, 12, or 24 h.

### Cell Cytotoxicity LDH Test

The impact of each SCFA on the cell cytotoxicity was assessed by LDH and the procedure described by the manufacturer followed. Briefly, 100 μl cell suspension (0.5–2 × 10^4^) was added into 96-well plates and medium was refreshed every 2–3 days until confluence. Cells were treated with acetate (1, 10, and 100 mM), butyrate (0.1, 0.3, and 1 mM) and propionate (0.3, 1, and 5 mM) for 48 h, and butyrate (5 mM) and propionate (10 mM) for 32 h. After that, the supernatants were collected and put into a new 96-well plate for LDH assays. 100 μl of the reaction mixture was added to each well and incubated in the dark at room temperature for 30 min before the absorbance of samples was measured at 490 nm. 1% Triton-X treated cells, which were killed 100%, were regarded as a positive control in comparison with other groups in the LDH analysis.

### Enzyme Linked Immunosorbent Assay (ELISA)

After pre-incubation with acetate, butyrate or propionate and LPS or TNFα stimulation, the supernatants were collected and stored at -20°C for further analysis. The levels of IL-6 and IL-8 in each group were measured by using ELISA according to the manufacturer’s instructions.

### VCAM-1 and ICAM-1 Expression

After pre-incubation with acetate (10 mM), butyrate (5 mM) and propionate (10 mM) for 24 h followed by 8 h LPS (1 μg/ml) or TNFα (1 ng/ml) stimulation, cells were detached from the cell culture plate surface by incubation with 0.05% trypsin/EDTA for 5 min at 37°C. Cell suspensions were centrifuged at 2151 g/min at 4°C for 5 min. Supernatants were discarded and cell pellets were re-suspended in PBS/0.1%BSA (w/v). PE-labeled ICAM-1 and VCAM-1 antibodies were added in a dilution of 1:800 (v/v) and cells were incubated for 30 min on the ice in the dark followed by staining with viability fixable dyes for live and dead cells analysis. Flow cytometry analyses were performed using BD FACS Canto II cytometer and Flowlogic version 7.

### Blood Mononuclear Cells Isolation

Human blood mononuclear cells from healthy donors were isolated from buffy coats (Sanquin, Amsterdam, Netherlands). Cells were purified using Ficoll-Paque Plus gradient centrifugation. (de Kivit et al., 2011) Briefly, mononuclear cells were isolated by centrifugation according to the manufacturer’s instructions. Mononuclear cells above the polyester gel were collected, washed with PBS containing 2% FBS by centrifuging at 1200 rpm for 5 min, re-suspended the cell pellet at a concentration of 2 × 10^6^cells/ml in RPMI1640 medium without phenol red containing 10% FBS and 1% penicillin-streptomycin. The viability of mononuclear cells was determined by trypan blue staining and cell number was counted.

### Endothelial Cell Monolayer Adhesion Experiment

Human umbilical vein endothelial cells were seeded in 96-well plates until they were confluent. Isolated mononuclear cells were washed with warm PBS three times and mononuclear cells (2 × 10^6^/mL) labeled with 1 μM calcein-AM for 30 min according to the manufacturer’s instruction. HUVEC were pre-incubated with propionate (10 mM) and butyrate (5 mM) for 24 h, followed by 8 h of TNFα stimulation. After stimulation, the medium was exchanged for fresh medium and co-cultured with labeled mononuclear cells (2 × 10^5^cells/well) for 30 min. The un-adhesive mononuclear cells were then washed away and HUVEC with adhesive mononuclear cells were fixed with 4% paraformaldehyde. The fluorescence image of labeled mononuclear cells was captured by Yokogawa CV7000S imager and relative fluorescence intensity was measured by Fluoroskan Ascent^TM^ FL with excitation wavelength 492 nm and emission wavelength 518 nm.

### Statistical Analysis

All data are expressed as mean ± standard deviation (SD). Group comparisons were performed using the one-way ANOVA analysis of variance of the experiments. The method of least-significant difference (LSD) was used as a *post hoc* test for multiple comparisons, to determine significant difference between specific treatment groups. In all cases, *P-value* < 0.05 was considered statistical significant.

## Results

### LPS or TNFα-Induced Release of IL-6 and IL-8 by HUVEC

Both LPS (1 or 10 μg/ml) and TNFα (100 pg/ml or 1 ng/ml) significantly enhanced the IL-6 and IL-8 release by HUVEC after 6, 12, and 24 h stimulation (**Supplementary Figure S1**). Based on these experiments, LPS and TNFα were, respectively, used at concentrations of 1 μg/mL and 1 ng/mL, and both incubations ran for 12 and 24 h in the experiments described in this manuscript.

### Basal Cytokine Release

The SCFA used in this experiment did not affect the non-stimulated (basal) cytokines level (data not shown). Besides, the concentrations of butyrate (0.1 mM) and propionate (0.3 mM) used in ELISA assays and butyrate (5 mM) and propionate (10 mM) used in flow cytometry assays were not toxic for HUVEC (**Supplementary Figure S2**).

#### The Optimal Concentrations of SCFA and Stimulation Time Duration

The optimal concentrations of the three SCFA for inhibition of IL-6 and IL-8 release by LPS or TNFα were: 0.1 mM for butyrate, 0.3 mM for propionate and 10 mM for acetate (data not shown). For acetate a pre-incubation time of 1, 16, and 24 h was most effective with 12 h LPS/TNFα stimulation, whilst a 24 h pre-incubation time with 1, 16, and 24 h stimulation times were chosen for butyrate and propionate. Under these experimental conditions, the most pronounced anti-inflammatory effects of SCFA were found (data not shown).

#### The Effects of Acetate on IL-6 and IL-8 Release

**FIGURE 1:**
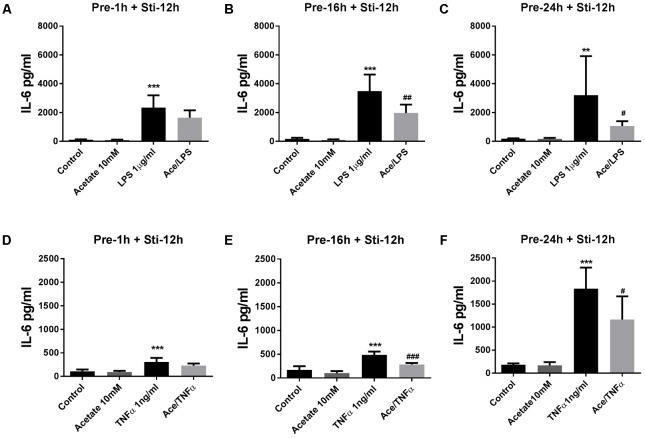
Acetate inhibited IL-6 release by HUVEC. Data show that pre-treatment of HUVEC with acetate (10 mM) for 1, 16, and 24 h reduced IL-6 stimulated by LPS 1 μg/mL **(A–C)** or TNFα 1 ng/mL **(D–F)** for 12 h. *N* = 4, ^∗∗^*p* < 0.01, ^∗∗∗^*p* < 0.001 compared with control group. ^#^*p* < 0.05, ^##^*p* < 0.01, ^###^*p* < 0.001 compared with LPS or TNFα group.

**FIGURE 2:**
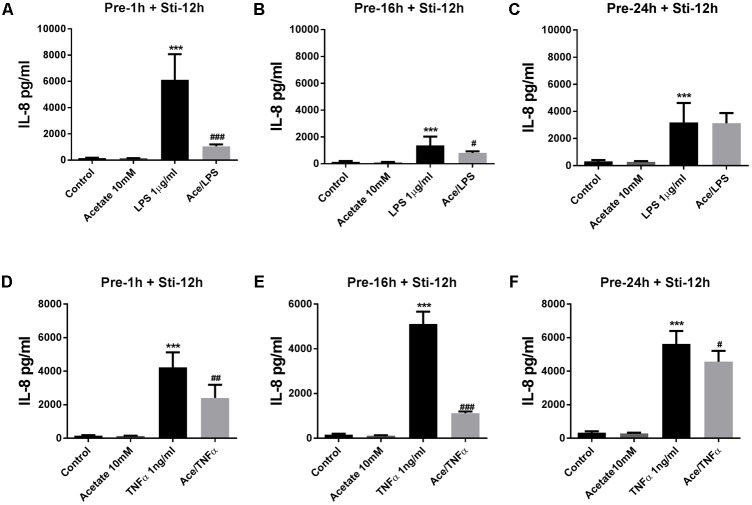
Acetate inhibited IL-8 release by HUVEC. Data show that pre-treatment of HUVEVC with acetate (10 mM) for 1, 16, and 24 h reduced IL-8 production stimulated by LPS 1 μg/mL **(A–C)** or TNFα 1 ng/mL **(D–F)** for 12 h. *N* = 4, ^∗∗∗^*p* < 0.001 compared with control group. ^#^*p* < 0.05, ^##^*p* < 0.01, ^###^*p* < 0.001 compared with LPS or TNFα group.

Pre-incubation in acetate for 16 or 24 h, but not for 1 h, significantly attenuated IL-6 after LPS stimulation (**Figures 1A–C**) and TNFα stimulation (**Figures 1D–F**). In the LPS-stimulated groups, IL-8 was significantly inhibited by acetate after 1 and 16 h pre-incubation (**Figures 2A,B**), but not after 24 h pre-incubation (**Figure 2C**). IL-8 production was inhibited after all pre-incubation times after TNFα-stimulation (**Figures 2D–F**).

#### The Effect of Butyrate on IL-6 and IL-8 Concentrations

**FIGURE 3:**
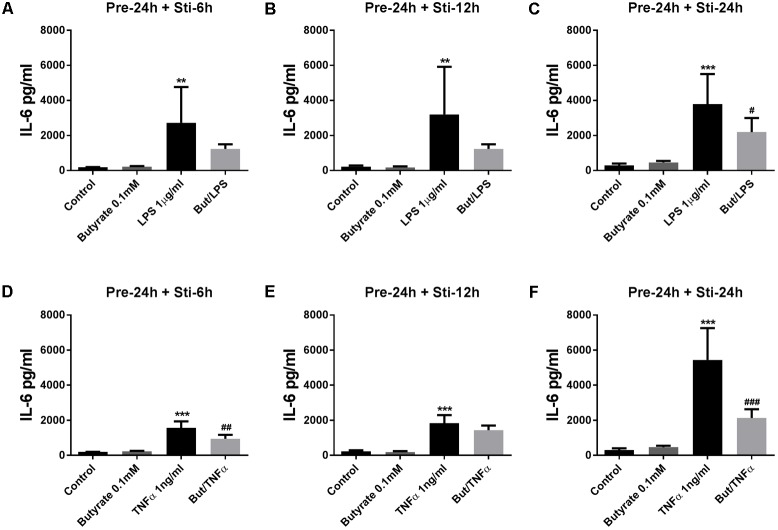
Effects of butyrate on IL-6 release. **(A–F)** The effects of 0.1 mM butyrate on IL-6 release with 24 h pre-incubation and 6, 12, or 24 h stimulation. **(A–C)** Butyrate combined with LPS. **(D–E)** Butyrate combined with TNFα. *N* = 4, ^∗∗^*p* < 0.01, ^∗∗∗^*p* < 0.001 compared with control group. ^#^*p* < 0.05, ^##^*p* < 0.01, ^###^*p* < 0.001 compared with LPS or TNFα group.

Butyrate (24 h pre-incubation) significantly attenuated IL-6 which reached significance in 24 h LPS stimulation group (**Figure 3C**), and butyrate also decreased IL-6 production in 6 and 12 h LPS stimulation groups (**Figures 3A,B**). With TNFα stimulation for 6 and 24 h, IL-6 concentration was significantly decreased (**Figures 3D,F**) and a decrease was observed after 12 h TNFα stimulation (**Figure 3E**).

**FIGURE 4:**
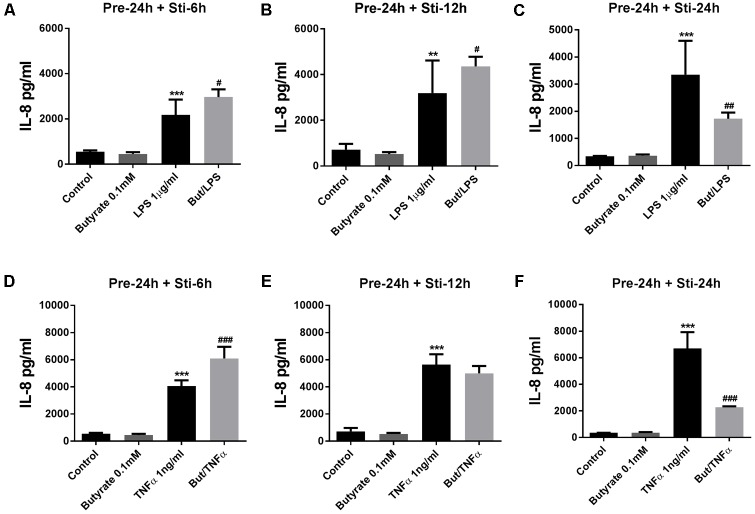
Effects of butyrate on IL-8 release. **(A–F)** 0.1 mM butyrate inhibited IL-8 release after 24 h pre-incubation and 6, 12, or 24 h stimulation. **(A–C)** Butyrate combined with LPS. **(D–F)** Butyrate combined with TNFα. *N* = 4, ^∗∗^*p* < 0.01, ^∗∗∗^*p* < 0.001 compared with control group. ^#^*p* < 0.05, ^##^*p* < 0.01,^###^*p* < 0.001 compared with LPS or TNFα group.

IL-8 (24 h pre-incubation) was significantly decreased by butyrate after 24 h LPS-stimulation (**Figure 4C**). In contrast, IL-8 was significantly increased after 6 and 12 h LPS stimulation compared to controls (**Figures 4A,B**). In the TNFα stimulated groups, butyrate increased IL-8 after 6 h TNFα stimulation (**Figure 4D**), and decreased IL-8 after 24 h TNFα stimulation (**Figure 4F**). No effect on IL-8 levels was observed after 12 h TNFα stimulation (**Figure 4E**).

#### The Effects of Propionate on IL-6 and IL-8 Release

**FIGURE 5:**
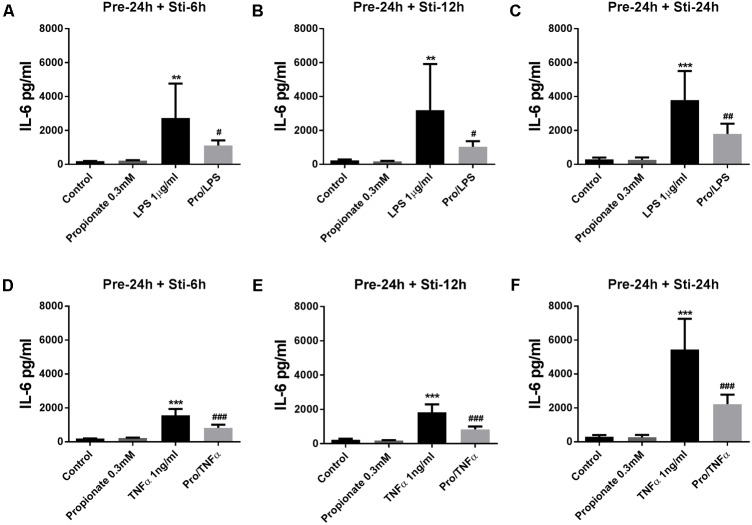
Effects of propionate on IL-6 release. **(A–F)** 0.3 mM propionate inhibited IL-6 release after 24 h pre-incubation and 6, 12, or 24 h stimulation. **(A–C)** Propionate combined with LPS. **(D–F)** Propionate combined with TNFα. *N* = 4, ^∗∗^*p* < 0.01, ^∗∗∗^*p* < 0.001 compared with control group. ^#^*p* < 0.05, ^##^*p* < 0.01,^###^*p* < 0.001 compared with LPS or TNFα group.

**FIGURE 6:**
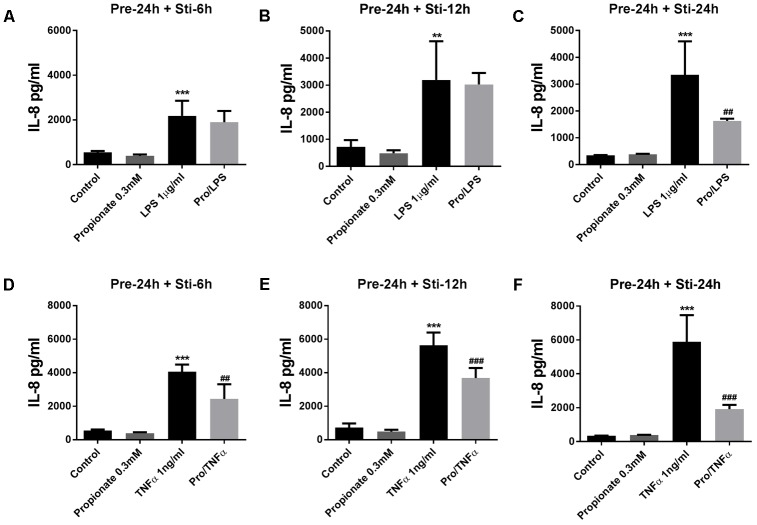
Effects of propionate on IL-8 release. **(A–C)** Propionate combined with LPS. **(D–F)** Propionate combined with TNFα. *N* = 4, ^∗∗^*p* < 0.01, ^∗∗∗^*p* < 0.001 compared with control group. ^##^*p* < 0.01,^###^*p* < 0.000 compared with LPS or TNFα group.

Propionate (24 h pre-incubation) significantly attenuated IL-6 production after LPS or TNFα stimulation (**Figure 5**). Propionate significantly inhibited IL-8 production after 24 h LPS stimulation but not after 6 and 12 h (**Figures 6A–C**). However, propionate reduced IL-8 at all time-points after TNFα stimulation (**Figures 6D–F**).

### Adhesion Molecules

The effects of SCFA on LPS- and TNFα-induced expression of ICAM-1 and VCAM-1 were examined since these adhesion molecules are essential for adhesion and diapedesis of inflammatory cells. In a pilot study, butyrate (0.1 mM) and propionate (0.3 mM) showed no effects on ICAM-1 and VCAM-1 expression (data not shown), therefore, butyrate (5 mM) and propionate (10 mM) were used to investigate the effects on adhesion molecules expression.

#### LPS- and TNFα-Induced Increase Expression of ICAM-1 and VCAM-1

On unstimulated HUVEC, ICAM-1 was expressed more than VCAM-1. Expression of both adhesion molecules were enhanced by LPS and TNFα stimulation (**Supplementary Figures S3A,B**).

ICAM-1 expression was significantly increased after 8 h of LPS or TNFα stimulation, and lasted for 24 h (**Supplementary Figure S3A**). VCAM-1 expression was significantly increased after 8 and 24 h LPS and 8 h of TNFα stimulation (**Supplementary Figure S3B**). Therefore 8 h stimulation time was used in the following experiments.

#### The Effects of SCFA on ICAM-1 and VCAM-1 Expression

**FIGURE 7:**
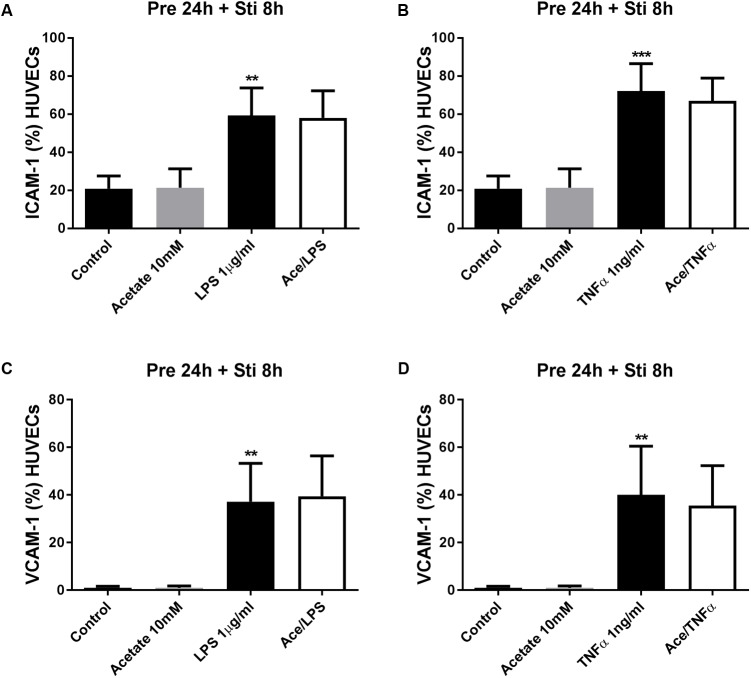
The effects of acetate on LPS- and TNFα-induced ICAM-1 and VCAM-1 expression. Acetate showed no effects on LPS- and TNFα- induced ICAM-1 **(A,B)** and VCAM-1 **(C,D)** expression. *N* = 3 ^∗∗^*p* < 0.01, ^∗∗∗^*p* < 0.001 compared with control group.

**FIGURE 8:**
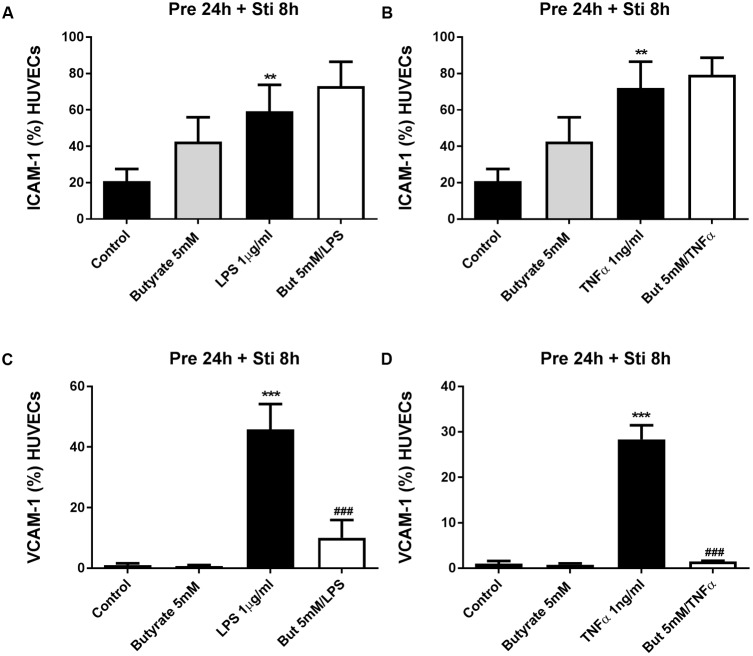
The effects of butyrate on LPS- and TNFα-induced ICAM-1 and VCAM-1 expression. **(A,B)** The effects of butyrate on LPS or TNFα induced ICAM-1 expression. **(C,D)** The effects of butyrate on LPS or TNFα induced VCAM-1 expression. *N* = 3, ^∗∗^*p* < 0.01, ^∗∗∗^*p* < 0.001 compared with control group. ^###^*p* < 0.001 compared with LPS or TNFα group.

**FIGURE 9:**
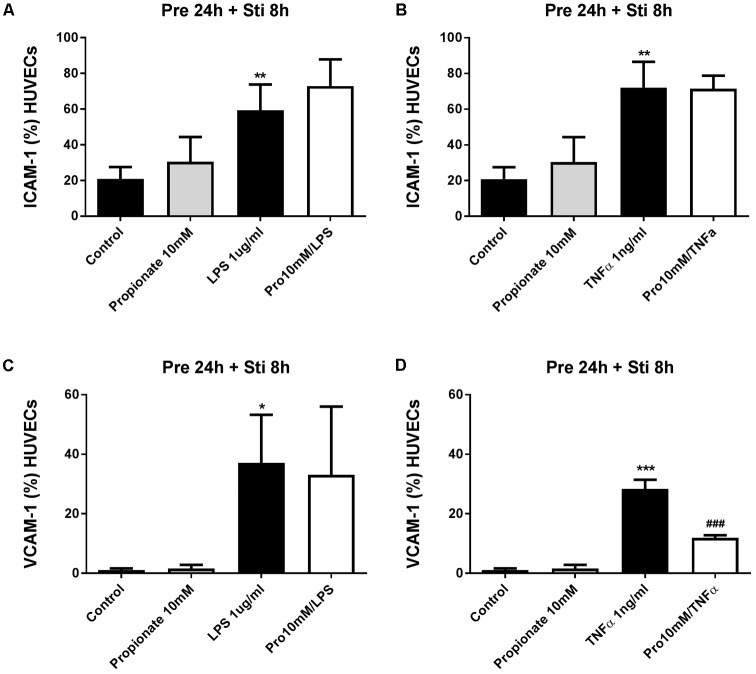
The effects of propionate on LPS- and TNFα induced VCAM-1 expression. **(A,B)** The effects of propionate on LPS or TNFα induced ICAM-1 expression. **(C,D)** The effects of propionate on LPS or TNFα induced VCAM-1 expression. *N* = 3, ^∗^*p* < 0.05, ^∗∗^*p* < 0.01, ^∗∗∗^*p* < 0.001 compared with control group. ^###^*p* < 0.001 compared with LPS or TNFα group.

Human umbilical vein endothelial cells were pre-treated with acetate (10 mM) for 24 h, followed by 8 h LPS or TNFα stimulation. Acetate did not affect ICAM-1 and VCAM-1 expression (**Figure 7**). Compared to the control group, the expression of ICAM-1 and VCAM-1 was increased after stimulation with LPS or TNFα. Butyrate profoundly inhibited VCAM-1 expression but not ICAM-1 (**Figures 8A–D**). Propionate did not affect the LPS- or TNFα-induced ICAM-1 expression (**Figures 9A,B**) and LPS-induced VCAM-1 expression (**Figure 9C**), but did reduced the TNFα-induced VCAM-1 expression (**Figure 9D**).

### Adhesion of Mononuclear Cells to Endothelial Cells

**FIGURE 10:**
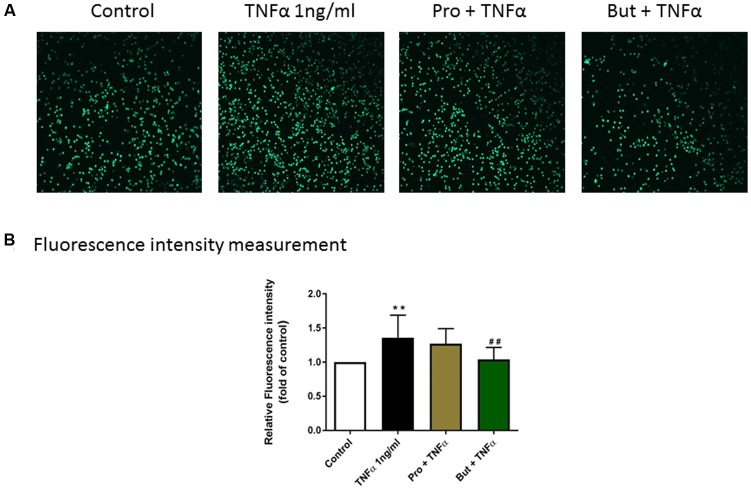
The functional role of butyrate and propionate on mononuclear cell adhesion to HUVEC monolayers. **(A)** Adhesive mononuclear cells were visualized by Yokogawa CV7000S imager. **(B)** The mean data of relative fluorescence intensity of adhesive mononuclear cells presented as the fold of control. *N* = 8. ^∗∗^*p* < 0.01 compared with control group; ^##^*p* < 0.01compared with TNFα group.

To investigate the functional role of propionate and butyrate on mononuclear cells adhesion to vascular endothelium, we conducted an adhesion assay by co-culturing mononuclear cells and HUVEC. The adhesion of mononuclear cells to HUVEC was significantly increased by TNFα (1 ng/ml) which was significantly inhibited by butyrate. A similar tendency was observed for propionate, although it was less effective than butyrate (**Figure 10**).

## Discussion

Endothelium is important in the development of inflammatory diseases including atherosclerosis (Hadi et al., 2005) and SCFA show potential roles in regulation of inflammation (Vinolo et al., 2011b), however, the evidence on the effects of SCFA on endothelial activation are controversial and incomplete. Hence, in the present study, we used three different SCFA (acetate, butyrate, and propionate), at various concentrations and incubation times to determine their effects on LPS or TNFα-induced endothelial activation including cytokines production and adhesion molecules expression. It is the first time to uncover the effects of SCFA on IL-6 and IL-8 production on LPS or TNFα-stimulated HUVEC and to investigate the effects of SCFA on ICAM-1 and VCAM-1 expression under this experimental set-up. In the current study, SCFA demonstrated a variety of effects on cytokine (IL-6 and IL-8) release depending on the condition chosen. Moreover, butyrate and propionate, but not acetate, inhibited VCAM-1 expression without changing ICAM-1 expression.

Previous studies demonstrated that treatment of butyrate reduced IL-6 production in oxLDL treated endothelial cell line (EA.hy926) (Aguilar et al., 2014). Thus, we expected that SCFA might inhibit cytokines production in LPS or TNFα-induced primary endothelial cells. Interestingly, we found SCFA reduced, increased or had no effects on the levels of IL-6 and IL-8 which depended on concentrations of SCFA used and pre-incubation/stimulation time applied. For example, pre-incubation with butyrate (0.1 mM) for 1 h increased IL-6 production, however, pre-incubation for 24 h decreased IL-6 production. Or pre-treatment of acetate (10 mM) for 12 h decreased IL-6 production, while 100 mM acetate increased IL-6 level under the same experiment set-up (data not shown). Similar effects of SCFA were found in peripheral blood monocytes and neutrophils: both acetate and butyrate, but not propionate, induced IL-8, IL-6, and IL-1β cytokine release when these cells were exposed to high levels (20 mM) while lower levels (≤2 mM) did not result in a significant increase in cytokine production (Mirmonsef et al., 2012). These findings indicate that SCFA can have a biphasic effect on inflammation and different SCFA can have different effects. However, in the present study, we only focused on the anti-inflammatory effects of SCFA and tried to find regularity in the effects of each SCFA on IL-6 and IL-8 production. With 12 h LPS- or TNFα-stimulation, acetate (10 mM) inhibited or showed a tendency to decrease IL-6 and IL-8 production regardless of pre-incubation time. While for butyrate (0.1 mM) and propionate (0.3 mM), a pre-incubation period of 24 h was needed to decrease IL-6 and IL-8. Butyrate and propionate showed similar effects on cytokine production but effects of acetate were different. The discrepancies between the SCFA might be due to different potencies on receptors or different duration of signaling pathways. For example, acetate can activate the G-protein coupled receptor 41/43 (GPR41/43, also known as FFA3/2 receptor), while butyrate and propionate can both activate FFA2/3 receptors and inhibit HDACs (Miyoshi et al., 2008). Besides, their potencies on activation of FFA receptors and inhibition of HDACs are different. The potency orders of SCFA for FFA2 receptor is acetate ∼ propionate > butyrate, whereas for the FFA3 receptor this is propionate ∼ butyrate > acetate (Miyoshi et al., 2008; Ulven, 2012). In addition, butyrate is found to be the most potent HDAC inhibitor, propionate is less potent and acetate does not have HDAC inhibitory property (Waldecker et al., 2008). These properties might explain the differences in effects between acetate and butyrate/propionate.

Furthermore, adhesion molecules-promoted adhesive interactions between leukocytes and endothelial cells and transmigration into the site of injury (Muller, 2013) are crucial steps in inflammation, immunity and atherosclerosis. On resting endothelial cells, ICAM-1 is usually expressed at low levels, while VCAM-1 is either absent or present in very low levels (Jersmann et al., 2001). ICAM-1 and VCAM-1 expression was up-regulated at 8 h after stimulation by either LPS or TNFα. However, the effects of SCFA on ICAM-1 and VCAM-1 expression on endothelial cells reported in previous studies are conflicting. Some found that butyrate increased adhesion molecules expression on HUVEC (Miller et al., 2005) but others observed inhibitory effects on expression of both ICAM-1 and VCAM-1 (Zapolska-Downar et al., 2004). In this study, acetate had no effects on ICAM-1 and VCAM-1 expression. Propionate only inhibited TNFα-induced VCAM-1 expression, while butyrate selectively inhibited LPS and TNFα-induced VCAM-1 expression but not ICAM-1 expression which is in agreement with effects of butyrate on TNFα- or IL1- induced adhesion molecule expression on human vascular endothelial cells (Menzel et al., 2004; Zapolska-Downar et al., 2004). The discrepancy between acetate and butyrate/propionate might be due to their inhibitory properties on HDAC: acetate does not inhibit HDAC activity and butyrate is more potent than propionate (Vinolo et al., 2011a). Moreover, the effect of butyrate on VCAM-1 expression was more potent than propionate, which was similar as their inhibitory effects on HDAC activity (Miyoshi et al., 2008).Therefore, it can be speculated that the inhibition of VCAM-1 expression by butyrate and propionate might be regulated by HDACs. There was also discrepancy among the effects of butyrate/propionate on ICAM-1 and VCAM-1expression, which were regulated by different gene-specific signal transduction routes (Zapolska-Downar et al., 2004). Previous studies showed that TSA, an HDAC inhibitor, had no effect on TNF-α induced p65/p50 binding to a consensus NF-κB binding probe that plays an important role in ICAM-1 expression (Wang et al., 2007). However, TSA could significantly reduce VCAM-1 expression as TSA might inhibit transcription factors, such as endothelial interferon-regulatory factor 1 and GATA instead of NF-κB, that are also involved in regulating VCAM-1 expression in activated HUVEC (Wang et al., 2007). In our study, butyrate and propionate selectively inhibited VCAM-1 expression and VCAM-1 is more important in facilitating monocyte adherence (Menzel et al., 2004). Therefore, decreased VCAM-1 expression by butyrate/propionate might break the vicious cycle between cytokines production and immune cell adhesion and inhibit chronic inflammatory responses. In order to investigate the functional effects of butyrate and propionate on cell adhesion, human mononuclear cells were co-cultured with HUVEC monolayer. Mononuclear cells adhesion to endothelial cells was significantly inhibited by butyrate. Propionate was less effective than butyrate which might be the consequence of less pronounced suppression of VCAM-1 expression. By collecting these data, we have demonstrated a role for VCAM-1 in cell adhesion and that possible damage induced by mononuclear cells in cardiovascular diseases can be reduced by SCFA.

## Conclusion

Endothelial activation, characterized by excessive cytokines (IL-6 and IL-8) production and adhesion molecules (ICAM-1 and VCAM-1) expression, is a crucial factor in the development of atherosclerosis and SCFA regulated endothelial activation in different ways. We found that the pre-incubation time and concentration used are important factors for each SCFA to show inhibitory effects on IL-6 and IL-8 production. Moreover, butyrate and propionate but not acetate inhibited VCAM-1 expression without affecting ICAM-1 expression. The different mechanisms including activation of GPR41/43 and inhibition of HDACs by which SCFA influence endothelial function might be one way to explain these results and elucidating the inhibitory pathways induced by SCFA might open a new era in the treatment of (chronic) inflammatory (cardiovascular) diseases.

## Author Contributions

ML performed the experiments, analyzed the data, and wrote the article. All authors were responsible for the study design, interpretation of the data, and helped to write and gave final approval of the final version of the manuscript.

## Conflict of Interest Statement

BvE and JG were employed by Danone Nutricia Research. The other authors declare that the research was conducted in the absence of any commercial or financial relationships that could be construed as a potential conflict of interest. The reviewer CF and handling Editor declared their shared affiliation.

## Supplementary Material

**FIGURE S1 |** IL-6 and IL-8 release by HUVEC stimulated with different concentrations of LPS or TNFα at different time points. **(A)** IL-6 and **(B)** IL-8 production with LPS stimulation. **(C)** IL-6 and **(D)** IL-8 production with TNFα stimulation. *N* = 4, ^∗^*p* < 0.05, ^∗∗^*p* < 0.01, ^∗∗∗^*p* < 0.001 compared with control group.

**FIGURE S2 |** The effects of different concentrations of acetate, butyrate and propionate on HUVEC. After 48 h incubation, cells toxicity was determined by using LDH assay **(A)**. Besides, high concentrations of butyrate 5 mM and propionate 10 mM were incubated for 32 h and cell toxicity was tested by LDH **(B)**. Cells treated with only medium were control groups and cells exposed to 1% Triton-X were used as positive control of cytotoxicity. *N* = 5–6, ^∗∗∗^*p* < 0.001 compared with control group.

**FIGURE S3 |** LPS and TNFα induced ICAM-1 and VCAM-1 expression. With LPS or TNFα stimulation for 8, 12, or 24 h, **(A)** ICAM-1 was significantly increased; **(B)** VCAM-1 expression was upregulated. Data was presented as percentage of positive population. *N* = 3, ^∗^*p* < 0.05, ^∗∗^*p* < 0.01 compared with control group.
