# Supplementary figures and images for: Time and Concentration Dependent Effects of Short Chain Fatty Acids on Lipopolysaccharide- or Tumor Necrosis Factor α-Induced Endothelial Activation
Source: Front Pharmacol. 2018 Mar 19;9:233. doi: 10.3389/fphar.2018.00233 (PMC5867315; doi:10.3389/fphar.2018.00233)

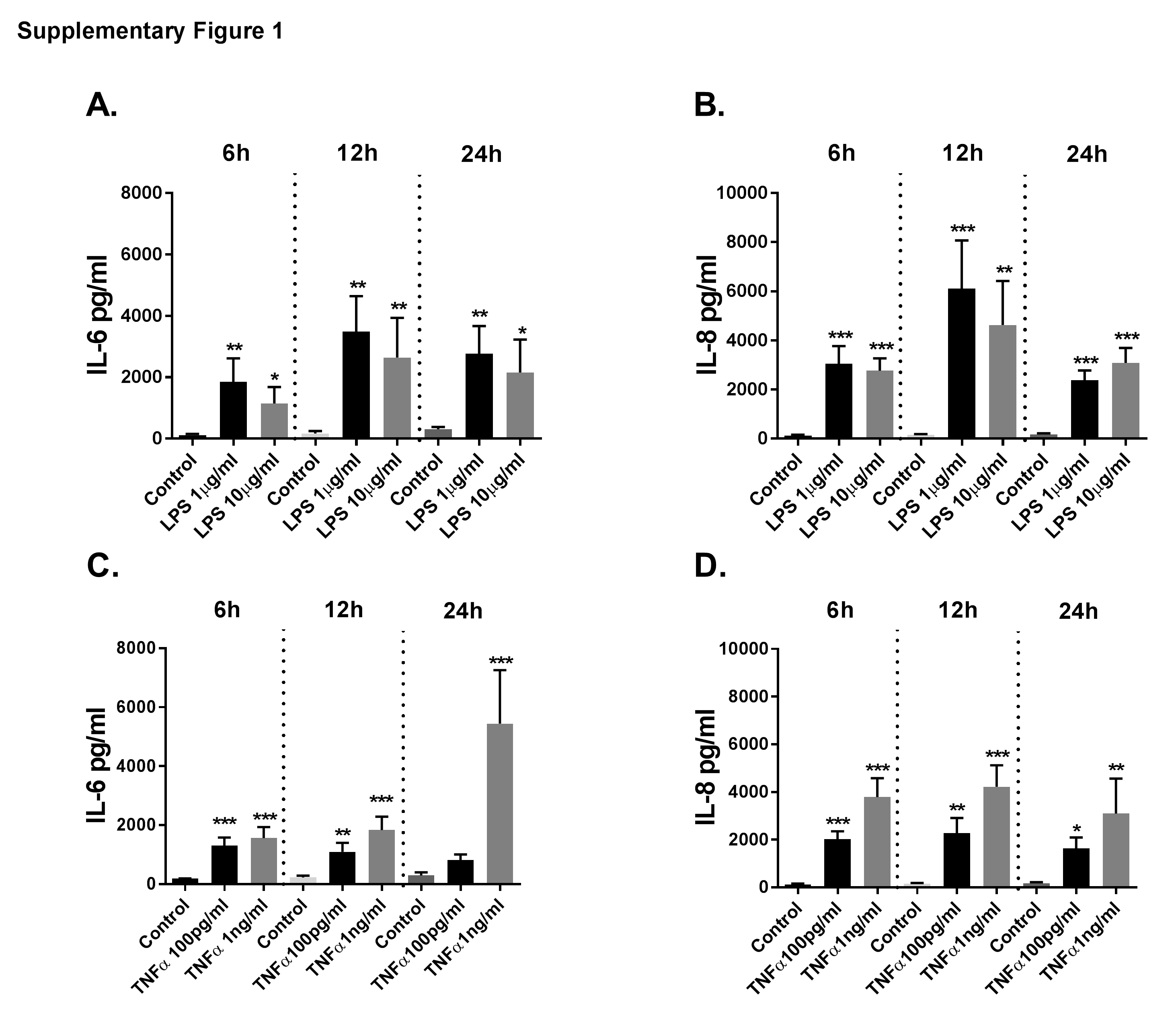

Supplement: Supplementary file 1 [file Image_1.TIF]

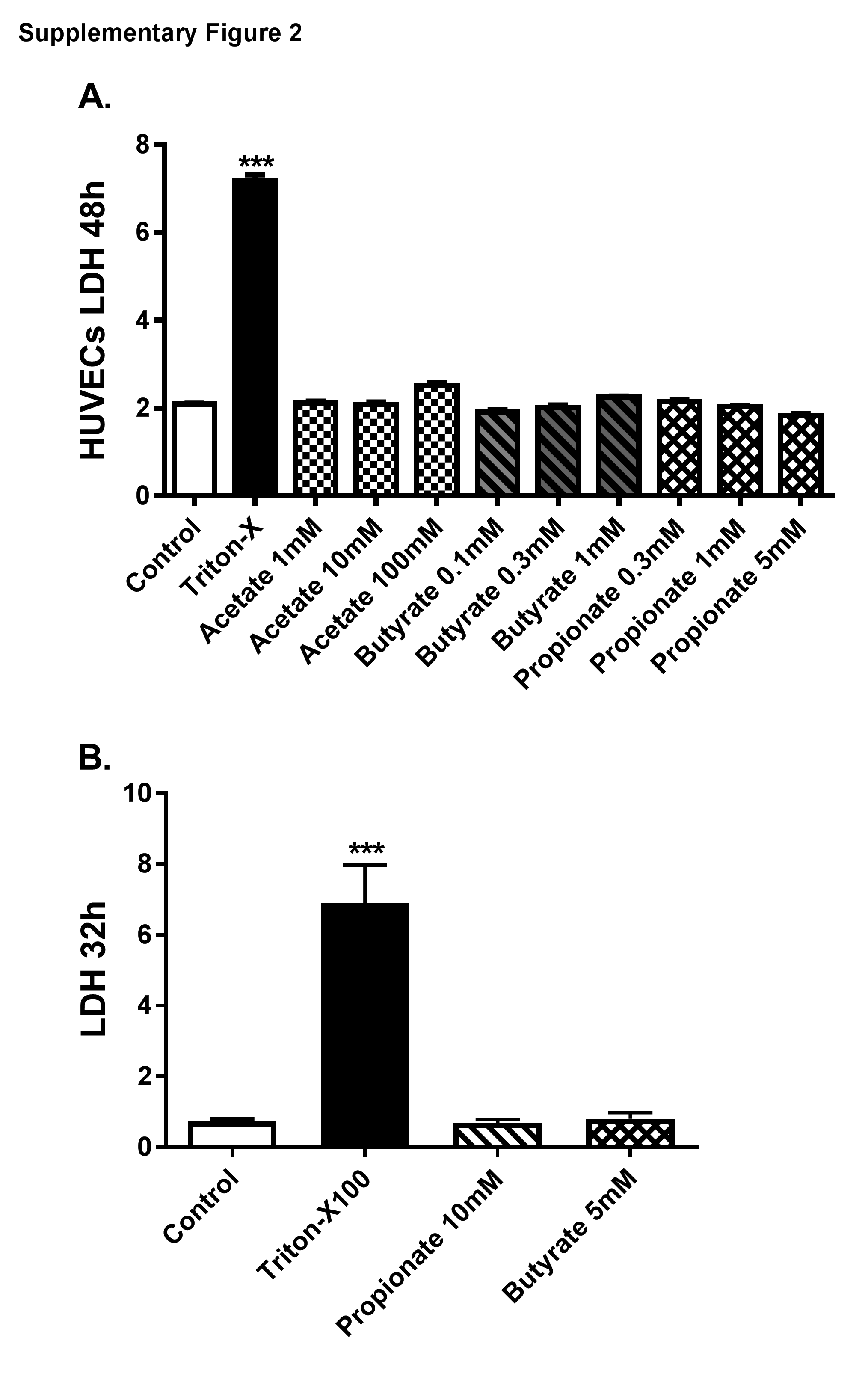

Supplement: Supplementary file 2 [file Image_2.TIF]

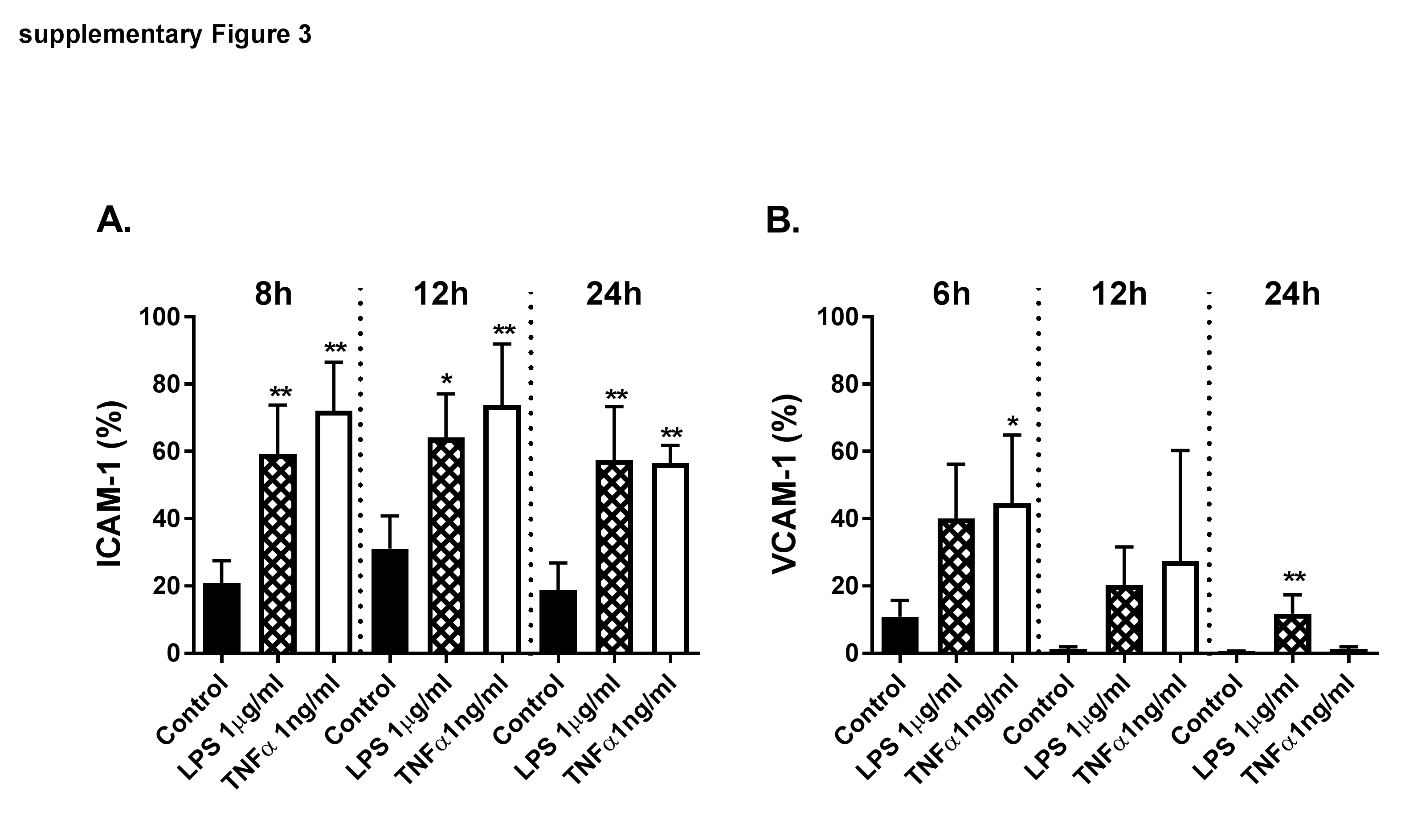

Supplement: Supplementary file 3 [file Image_3.TIF]
